# Biogeography and genetic diversity of clinical isolates of *Burkholderia pseudomallei* in Sri Lanka

**DOI:** 10.1371/journal.pntd.0009917

**Published:** 2021-12-01

**Authors:** Himali S. Jayasinghearachchi, Enoka M. Corea, Kumari I. Jayaratne, Regina A. Fonseka, Thilini A. Muthugama, Jayanthi Masakorala, Ravija YC. Ramasinghe, Aruna D. De Silva

**Affiliations:** 1 Institute for Combinatorial Advance Research and Education (KDU-CARE), General Sir John Kotelawala Defence University, Ratmalana, Sri Lanka; 2 Department of Medical Microbiology and Immunology, Faculty of Medicine, University of Colombo, Colombo, Sri Lanka; 3 Biomedical Laboratory 2, Faculty of Medicine, General Sir John Kotelawala Defence University, Ratmalana, Sri Lanka; 4 Department of Para-Clinical Sciences, Faculty of Medicine, General Sir John Kotelawala Defence University, Ratmalana, Sri Lanka; University of Florida, UNITED STATES

## Abstract

**Background:**

Melioidosis is a potentially fatal infectious disease caused by *Burkholderia pseudomallei* and the disease is endemic in Southeast Asia and Northern Australia. It has been confirmed as endemic in Sri Lanka. Genomic epidemiology of *B*. *pseudomallei* in Sri Lanka is largely unexplored. This study aims to determine the biogeography and genetic diversity of clinical isolates of *B*. *pseudomallei* and the phylogenetic and evolutionary relationship of Sri Lankan sequence types (STs) to those found in other endemic regions of Southeast Asia and Oceania.

**Methods:**

The distribution of variably present genetic markers [*Burkholderia* intracellular motility A (*bimA)* gene variants *bim*A_BP_/*bimA*_BM_, filamentous hemagglutinin 3 (*fhaB3)*, *Yersinia-*like fimbrial (YLF) and *B*. *thailandensis*-like flagellum and chemotaxis (BTFC) gene clusters and lipopolysaccharide O-antigen type A (LPS type A)] was examined among 310 strains. Multilocus sequence typing (MLST) was done for 84 clinical isolates. The phylogenetic and evolutionary relationship of Sri Lankan STs within Sri Lanka and in relation to those found in other endemic regions of Southeast Asia and Oceania were studied using e BURST, PHYLOViZ and minimum evolutionary analysis.

**Results:**

The Sri Lankan *B*. *pseudomallei* population contained a large proportion of the rare BTFC clade (14.5%) and *bimA*_BM_ allele variant (18.5%) with differential geographic distribution. Genotypes *fhaB3 and* LPSA were found in 80% and 86% respectively. This study reported 43 STs (including 22 novel). e-BURST analysis which include all Sri Lankan STs (71) resulted in four groups, with a large clonal group (group 1) having 46 STs, and 17 singletons. ST1137 was the commonest ST. Several STs were shared with India, Bangladesh and Cambodia.

**Conclusion:**

This study demonstrates the usefulness of high-resolution molecular typing to locate isolates within the broad geographical boundaries of *B*. *pseudomallei* at a global level and reveals that Sri Lankan isolates are intermediate between Southeast Asia and Oceania.

## Author summary

*Burkholderia pseudomallei* is an important cause of community acquired pneumonia, septicemia and abscesses in Sri Lanka. The risk of infection is increased after flooding following heavy rainfall. Risk groups include rice farmers and rural populations engaged in subsistence cultivation in home gardens. Nationwide surveillance has been carried out since 2006 and the state public health system offers free diagnostics and free antibiotic therapy. The incidence of melioidosis in Sri Lanka has increased in tandem with increased awareness among clinicians. This study reports the genetic diversity among Sri Lankan *B*. *pseudomallei* clinical isolates and shows that some variably present genes are regionally distributed. The population is intermediate between Southeast Asia and Oceania. This may reflect its past geological history.

## Introduction

*Burkholderia pseudomallei* is a soil-dwelling, Gram-negative, saprophytic environmental bacterium that causes the potentially fatal infectious disease, melioidosis [1–3]. The disease is considered an emerging threat throughout tropical and subtropical regions globally and is endemic in Southeast Asia and Northern Australia [2,4]. The bacterium can be found in soil and fresh water bodies in endemic regions and infection is acquired via percutaneous inoculation, inhalation or ingestion [1,2,4]. Melioidosis has recently been confirmed as endemic in Sri Lanka [5] and the risk is increased during heavy rains [6]. Agricultural workers have been identified as vulnerable because of frequent exposure to soil and water [1,5]. Clinical manifestations of melioidosis are greatly diverse regardless of the route of infection, from localized abscesses to severe pneumonia or life-threatening sepsis, and occasionally neurological melioidosis [1,7,8]. The reported case fatality rates are up to 40% and relapse rates are ∼20% in certain settings [1,9,10]. Successful antibiotic treatment has proven to be crucial for favorable outcomes in humans. There are no vaccines currently available. Therefore, the organism was upgraded to Tier 1 Select Agent characterization by US Centers for Disease Control and Prevention in 2012 [11,12]. A request has been submitted to make it a notifiable disease in Sri Lanka.

A broad strain diversity has been reported from endemic regions such as northern Australia and Southeast Asia [2,9]. Multilocus sequence typing (MLST), which is based on sequence variation in seven housekeeping genes, has been successfully employed as an epidemiological tool to characterize the genetic diversity of *B*. *pseudomallei* within and across endemic regions [13,2]. e-BURST analysis (based upon sequence typing) has shown the presence of discrete clonal complexes which are specific to each endemic region indicating population groupings [3,14,15]. However, shared sequence types (STs) and single locus variants (SLVs) are widely distributed in India, Australia, Thailand and China [2,16,17].

A small number of Sri Lankan clinical strains of *B*. *pseudomallei* has been characterized so far using MLST and whole genome sequence analysis [5,15,18]. However, more work needs to be done to explore the biogeography and genetic diversity of *B*. *pseudomallei* in Sri Lanka. Therefore, the main aim of the present study was to determine genetic diversity among Sri Lankan clinical isolates and their regional distribution using MLST and real-time polymerase chain reaction (RT-PCR) based genotyping methods. The distribution of variably present epidemiological markers [*Burkholderia* intracellular motility A (*bimA*) gene variants *bim*A_BP_/*bimA*_BM_, filamentous hemagglutinin 3 (*fhaB3)*, *Yersinia-*like fimbrial (YLF) gene cluster and *B*. *thailandensis*-like flagellum and chemotaxis (BTFC) gene cluster and lipopolysaccharide O-antigen type A (LPSA)] was examined among 310 clinical isolates while MLST was done for 84 and the STs of all isolates, including those previously reported from, Sri Lanka, were subjected to e-BURST analysis. The phylogenetic and evolutionary relationship of Sri Lankan STs within Sri Lanka and in relation to those found in other endemic regions of Southeast Asia and Oceania was studied.

## Methods

### Ethics statement

Ethics approval was obtained from the Ethics Review Committee, Faculty of Medicine, University of Colombo, Sri Lanka (EC-17-020). Written or verbal consent was not obtained in this study as all bacterial isolates and other information were used anonymously.

### Clinical isolates

A total of 310 consecutive clinical isolates of *B*. *pseudomallei* collected from melioidosis cases (one isolate per case) across the country from 2006 to early 2018 were included in this study. All isolates were confirmed as *B*. *pseudomallei* by latex agglutination and *lpxo* RT-PCR assays [19,20]. Patient demographic and geographic data including age, sex, address, occupation, clinical presentation, underlying risk factors and outcome were recorded. MLST analysis was limited to 84 strains due to financial constraints (MLST for 109 isolates was already available in the *B*. *pseudomallei* MLST database).

### Detection of variably present gene markers

Cryo-preserved bacterial cultures were grown overnight in blood agar medium at 35°C. Total genomic DNA was extracted from pure cultures using a commercially available bacterial genomic DNA extraction kit as per the manufacturer’s instructions (CEYGEN Biotech, Sri Lanka). The extracted DNA from each isolate was stored at -20°C until use. Optimized multiplex RT-PCR assays with gene specific oligonucleotides primers were performed to detect YLF and BTFC gene clusters and variably present genes *fhab3*, *bimA*_*Bp*_*/bimA*_*Bm*_ and LPSA [21–23]. BRYT green qRT-PCR master mixture (Promega, USA) was used in this study. Known positive DNA extracted from *B*. *pseudomallei* clinical isolates previously confirmed by whole genome sequencing were used as positive controls [24].

### MLST of 84 clinical isolates of *B*. *pseudomallei*

#### Amplification of housekeeping genes

Amplification of seven housekeeping genes (*ace-gltB-gmhD-lepA-lipA-narK-ndh*) was performed using established oligonucleotide primer sequences as reported previously [13], http://pubmlst.org/bpseudomallei.mlst.net/. Five microliters of PCR products were analyzed via 1.5% agarose gel electrophoresis to check for sufficient product, correct size and product purity.

#### Purification of amplicons and sequencing

PCR products were submitted for amplicon purification and SANGER sequencing to Macrogen Inc., Seoul, South Korea. Each DNA fragment was sequenced in forward and reverse directions using the same oligonucleotide primers that were used for the initial PCR amplification.

#### Sequence analysis and data submission to MLST database

For the sequence analysis, the forward and reverse sequences were aligned with a reference allele sequence obtained from the *B*. *pseudomallei* MLST website (http://pubmlst.org/bpseudomallei.mlst.net/) using Clustal Omega (https://www.ebi.ac.uk/Tools/msa/clustalo/). The sequences were edited by trimming to the appropriate size for each locus. The batch sequence (7 loci) from each isolate was queried in the MLST database to determine the allelic profile. The allelic profile of each isolate was queried for a match to the existing sequence types on the MLST database. The allele profile data for novel STs were submitted to the curator for confirmation and assignment of allelic numbers and STs. All isolate data and STs published in this study can be found at the *B*. *pseudomallei* MLST database (http://bpseudomallei.mlst.net/).

Multilocus sequence analysis (MLSA) was performed using e-BURST with SLV selected. A total of 193 isolate records with 71 STs reported from Sri Lanka (84 from this study and 109 submitted previously) was used for the e-BURST analysis and the criterion of six shared alleles out of seven alleles was used to delineate the clonal complexes [25]. Further, PHYLOVIZ 2.0 [26]. available at PubMLST (http://pubmlst.org/bpseudomallei.mlst.net/) was used to reveal the genetic relationship of STs within Sri Lanka and in relation to those found in Australia, India, Thailand, China, Cambodia, Malaysia and Vietnam.

### Construction of minimum-evolutionary tree (MET)

A minimum -evolutionary tree was constructed from the concatenated sequences (3401 positions) in the order of loci *ace-gltB-gmhD-lepA-lipA-narK-ndh* for Sri Lankan STs and those STs that showed SLVs and double locus variants (DLVs) to Sri Lankan STs. The evolutionary history was inferred using the Maximum Likelihood method [27]. The bootstrap method was used to test the phylogeny. The evolutionary distances were computed using the Tamura and Nei Model. The MET was searched using the Close-Neighbor-Interchange (CNI) algorithm [28] at a search level of one. The Neighbor-joining algorithm was used to generate the initial tree [29]. Evolutionary analyses were conducted in MEGA X [30]. Measure of genetic differentiation (F_ST_) was calculated for the Sri Lankan population using DNaSP Version: 6.12 [31].

### Association of *bimA*_BM_ genotype with neurological melioidosis

Association of *bimA*_BM_ and neurological melioidosis was determined using the Fisher’s exact test in GraphPad Prism 8.4.3. Neurological syndromes such as meningitis, encephalitis and myelitis were considered as neurological melioidosis but cerebral abscesses were excluded.

## Results

Melioidosis cases reported across Sri Lanka during 2006 to early 2018 (n = 310) comprised 215 (69%) males and 95 (31%) females, spanning an age range of 11 months to 98 years with a median of 51 years. Abscesses (with or without sepsis) were the most common clinical presentation with 48% (n = 150) of cases followed by pneumonia (29%, n = 89) and septic arthritis (15%, n = 47) (S1 Table). The case fatality rate was 24%, n = 75).

**Fig 1:**
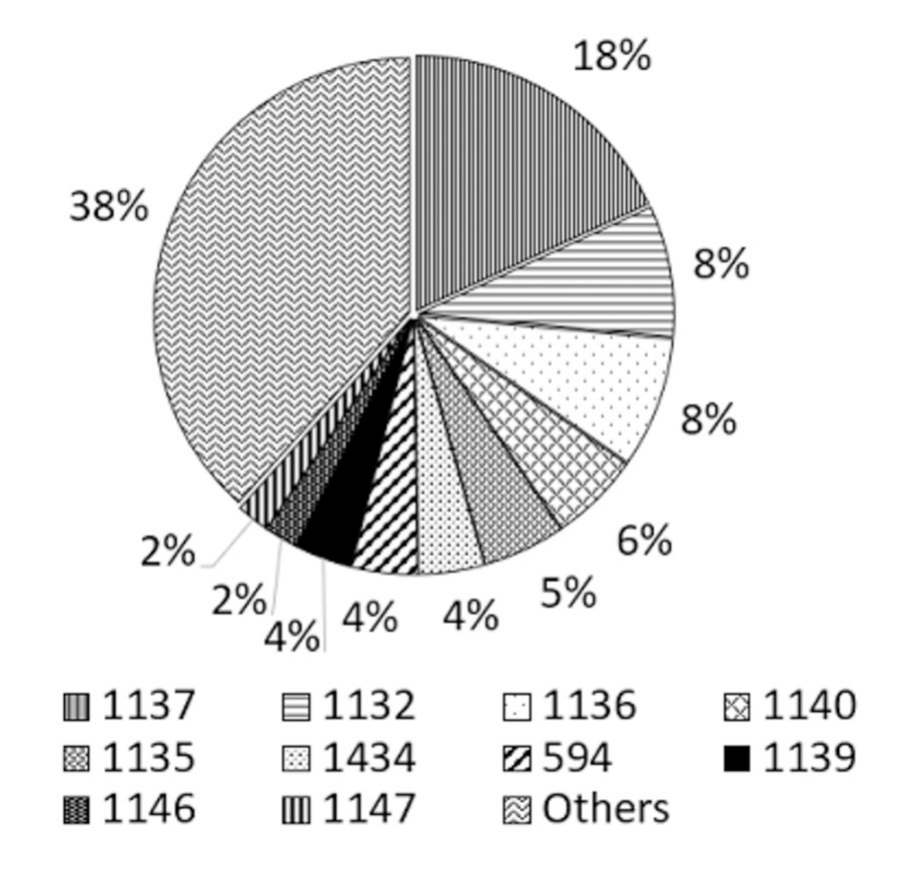
Percentage distribution of commonest STs.

Eighty percent (n = 248) were from rural settings. Main occupational groups included housewives (16%, n = 50) and rice farmers (15%, n = 45). The commonest underlying risk factor was diabetes (64%, n = 199). Percentage distribution of STs (n = 193) is shown Fig 1. Culture positive melioidosis cases per 100,000 population for each of the nine provinces is shown in Table 1.

**Table 1:** Culture positive melioidosis cases per 100,000 population for each of the nine provinces.

Province	No. of cases	Population (2012)	Cases/100,000 population
WP	103	6,165,000	1.67
NWP	58	2,380,861	2.44
EP	47	1,555,510	3.02
SP	41	2,477,285	1.66
CP	16	2,571,557	0.62
NCP	9	1,266,663	0.71
UVA	8	1,266,463	0.63
SG	8	1,928,655	0.41
NP	12	1,061,315	1.13

WP-Western Province, SP-Southern Province

EP-Eastern Province, NP-Northern Province, NWP-North Western Province, SGP-Sabaragamuwa Province, CP-Central Province, NCP-North Central Province.

**Fig 2:**
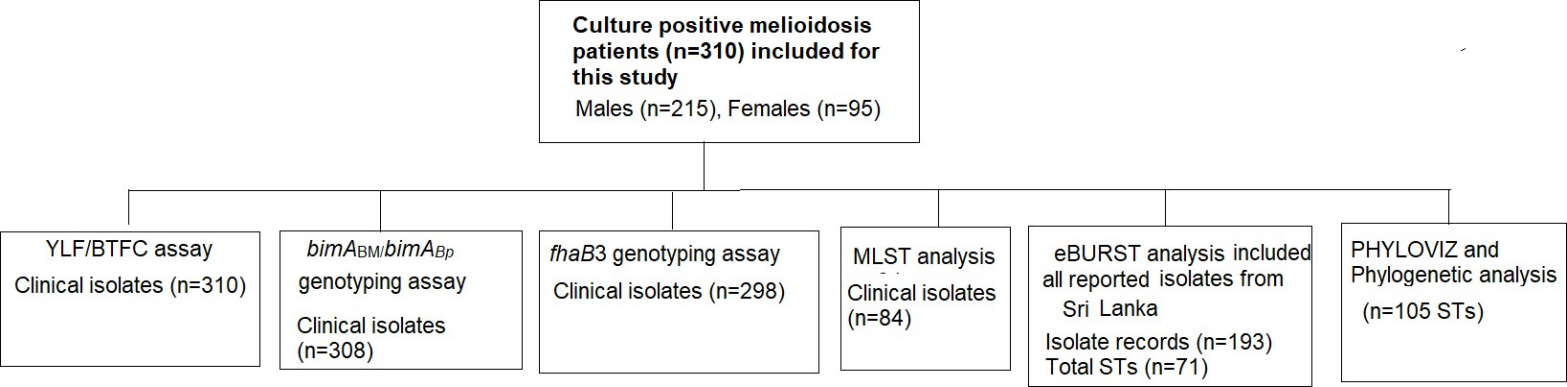
Flowchart demonstrating genotyping and analyses of clinical isolates.

Genotyping and analyses were performed as shown in Fig 2.

### Distribution of epidemiology markers YLF/BTFC, *bimA*, *fhaB3* and LPSA

**Fig 3:**
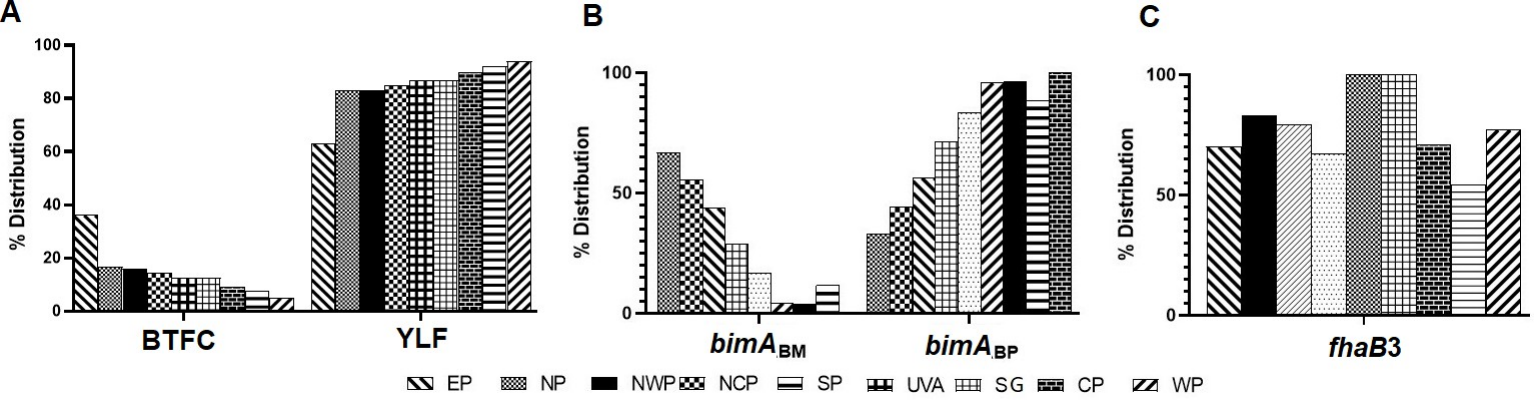
**A.** Distribution of genotypes of *Burkholderia pseudomallei* in 9 provinces in Sri Lanka. A. *Yersinia-*like fimbrial (YLF) and *B*. *thailandensis* -like flagellum and chemotaxis (BTFC) gene clusters, **B.** *Burkholderia* intracellular motility factor A (*bimA*_*BM*_ *bimA*_Bp_), **C.** Filamentous hemagglutinin 3 (*fhaB3*). WP-Western Province, SP-Southern Province, Eastern Province, NP-Northern Province, NWP-North Western Province, SGP-Sabaragamuwa Province, CP-Central Province, NCP-North Central Province.

Genotyping of clinical isolates (n = 310) showed that the majority of Sri Lankan isolates belong to the YLF group (85.5%, n = 265). However, about 14.5% (n = 45) of BTFC positive isolates was found. Further, this study examined the regional distribution of YLF/BTFC gene clusters within the country (Fig 3A and S2 Table). It was noted that the relative frequency of the BTFC group was found to vary among regional populations. Sri Lanka is administratively divided into 9 provinces and, while isolates with YLF gene cluster were seen in all provinces, isolates with BTFC gene cluster were predominantly present in the Eastern Province (36.17%, 17/47) (Fig 3A).

The rare allele variant *bimA*_BM_ was found in 18.5% (57/308) of the Sri Lankan *B*. *pseudomallei* population (S3 Table). Regional variation in its distribution was found with a predominance in the Northern, North Central and Eastern Provinces (Fig 3B). Among these, 19 isolates (33%) were in the BTFC clade.

As the literature has shown an association between neurological melioidosis and *bimA*_BM_, we explored this phenomenon in our population. Five of eleven (45.45%) neurological melioidosis cases were caused by isolates with genotype *bimA*_BM_ although this genotype consisted of only 57 of 308 (18.5%) available genotypes. A Fisher’s exact Test of independence showed that there was a significant association between *bimA*_BM_ genotype and neurological melioidosis with (1, n = 308) = *p* = 0.0345).

The majority (80%, 238/298) of Sri Lankan clinical *B*. *pseudomallei* isolates possessed the *fhaB3* variant. However, there was no regional preference in its distribution within the country (Fig 3C). Majority of Sri Lankan *B*. *pseudomallei* was found to carry the LPSA gene cluster (86%, 252/294). The four genotypes were randomly distributed among the strains showing no specific associations.

### MLST analysis

MLST analysis of 84 clinical isolates resulted in 43 STs, of which 22 were novel: ST 1880–85, ST1887-95, ST1898, ST1900, ST1928-30, ST1933-34 (S4 Table). Two distinct alleles *ace* 55, and *ndh* 124 were found to be unique to Sri Lanka and are shown in bold numbers (Table 2). Considering the total Sri Lankan isolates in the database (a total of 193 records with 71 STs), the dominant alleles were *ace*-1, *gltB*-2, *gmhD*-6, *lepA*-2, *lipA*-1, *narK*-2 and *ndh*-3 (Table 2). Alleles *gltB* and *ndh* were the most variable with 7 variants each while *ace* had only 5 variants. ST1137 was found to be the most abundant ST (n = 35 isolates) followed by ST1136 (n = 16) and ST1132 (n = 16). When regional distribution is considered, ST1132 concentrated in the NWP (62.5%, 10/16) while ST1137 concentrated in the WP (60%, 21/35) (S5–S7 Tables).

**Table 2:** Prevalence of alleles in the Sri Lankan population (n = 193) of *Burkholderia pseudomallei*.

Locus	Allele number and prevalence (%)
*ace*	1 (76.5), 4 (18.4), 8 (3.6), 18 (1.0), **55** (0.5)
*gltB*	2 (55.6), 4 (22.4), 12 (18.4), 1 (1.5), 5 (0.5), 6 (0.5), 16 (0.5)
*gmhD*	6 (64.3), 3 (19.4), 14 (9.2), 10 (3.1), 13 (3.1), **124** (0.5)
*lepA*	2 (74.5), 4 (21.9), 3 (1.0), 1 (1.0), 46 (0.5), 19 (0.5),
*lipA*	1 (86.2), 5 (7.1), 20 (3.1), 3 (2.0), 25 (1.0), 6 (1.0)
*narK*	2 (41.8), 1 (30.6), 8 (24.0), 42 (1.5), 60 (1.5), 21 (0.5)
*ndh*	3 (55.1), 1 (23.0), 57 (15.8), 20 (2.6), 3 (1.5), 11 (1.0), 87 (0.5),

### Genetic relatedness among STs from Sri Lanka

**Fig 4:**
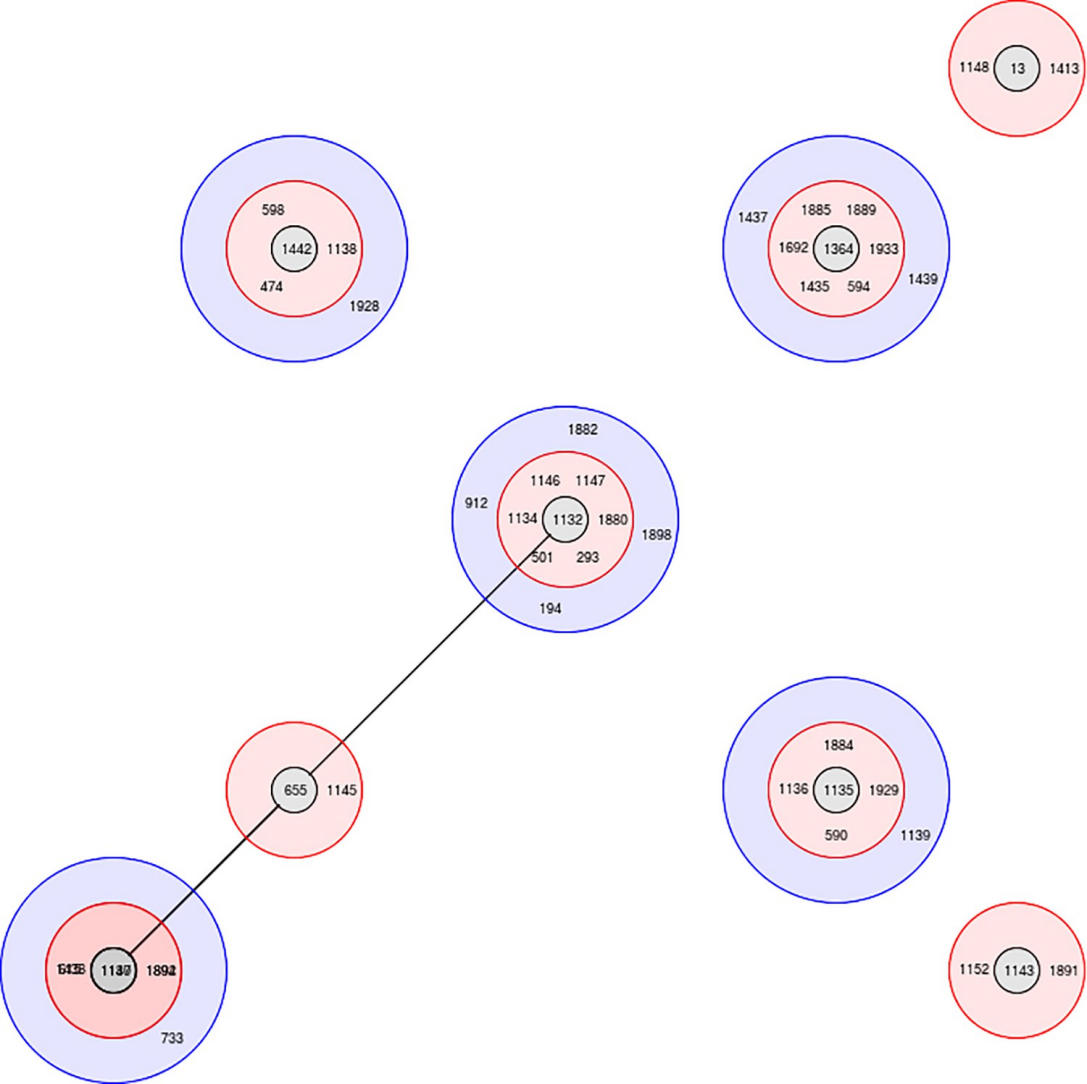
e-BURST tree showing the hypothetical pattern of descent for clinical isolates of *Burkholderia pseudomallei* in Sri Lanka. Red shade depicts single locus variants (SLVs) while blue shade depicts double locus variants (DLVs).

e-BURST analysis revealed four groups with a large clonal group (Group1) having 46 STs distributed among eight sub-group founders (Fig 4). Group 2, 3 and 4 comprised four, two and two STs respectively. Seventeen (17) singletons (outliers) were also seen (Table 3). According to the current data, ST1132 was the predicted founder which has 16 clinical isolates reported so far (S4 Table). Single locus variants were ST293, ST501, ST1134, ST1146, ST1147, ST1880 and double locus variant ST194, ST912, ST1882, ST1898.

**Table 3:** Description of the study isolates based on eBURST analysis.

**Group1**				
**Sequence type**	**Frequency**	**Single locus variant**	**Double locus variant**	**Satellites (more distantly related isolates)**
13	2	2	1	42
194	1	3	7	35
293	1	4	15	26
474	1	1	2	42
501	1	5	10	30
590	2	1	4	40
594	8	4	8	33
598	1	2	2	41
615	1	1	4	40
655	1	5	14	26
733	1	1	4	40
912	2	1	4	40
1132*	16	7	10	28
1134	1	3	8	34
1135	10	4	3	38
1136	16	3	5	37
1137	35	5	10	30
1138	1	2	6	37
1139	7	1	3	41
1140	12	4	5	36
1143	3	4	8	33
1145	1	5	12	28
1146	4	1	8	36
1147	4	6	9	30
1148	1	1	2	42
1152	2	2	7	36
1364	2	6	6	33
1413	1	2	5	38
1435	2	3	7	35
1437	1	2	10	33
1438	1	1	4	40
1439	2	1	3	41
1442	1	3	4	38
1692	1	5	9	31
1880	1	3	9	33
1882	1	2	7	36
1884	1	4	8	33
1885	1	2	6	37
1889	2	2	8	35
1891	1	2	2	41
1892	1	2	5	38
1894	1	1	7	37
1898	1	2	6	37
1928	1	1	1	43
1929	1	2	3	40
1933	1	2	7	36
**Group 2**				
867	1	2	1	
1179	1	1	2	
1888	1	1	2	
1934	1	2	1	
**Group 3**				
1434	8	1	0	
1436	1	1	0	
**Group 4**				
1893	1	1	0	
1900	1	1	0	
**Singletons:** 132, 202, 308, 338, 421, 944, 1133, 1141, 1142,1144, 1314,1881,1883, 1887,1890,1895,1930				

Shared STs observed were ST202, ST594 (Australia, Thailand), ST912 (Cambodia), ST501 (India, Thailand), ST912 (Cambodia and India), ST1692, ST293, ST1143, ST1152 (India), ST13, ST655, ST308 (Thailand), ST132 (Australia) and ST421 (Belgium). The shared STs ST501 and ST293 are SLVs of ST1132. The shared ST13 formed a subgroup founder with two SLVs ST1413 and ST1148 while shared ST655 was the subgroup founder of SLV ST1145 and was distantly related to ST1132 and the commonest ST1137. The shared ST1143 was found as a subgroup founder with shared ST1152 and the novel ST1891 (Fig 4).

### Phylogenetic relatedness of Sri Lankan STs with a global collection

**Fig 5:**
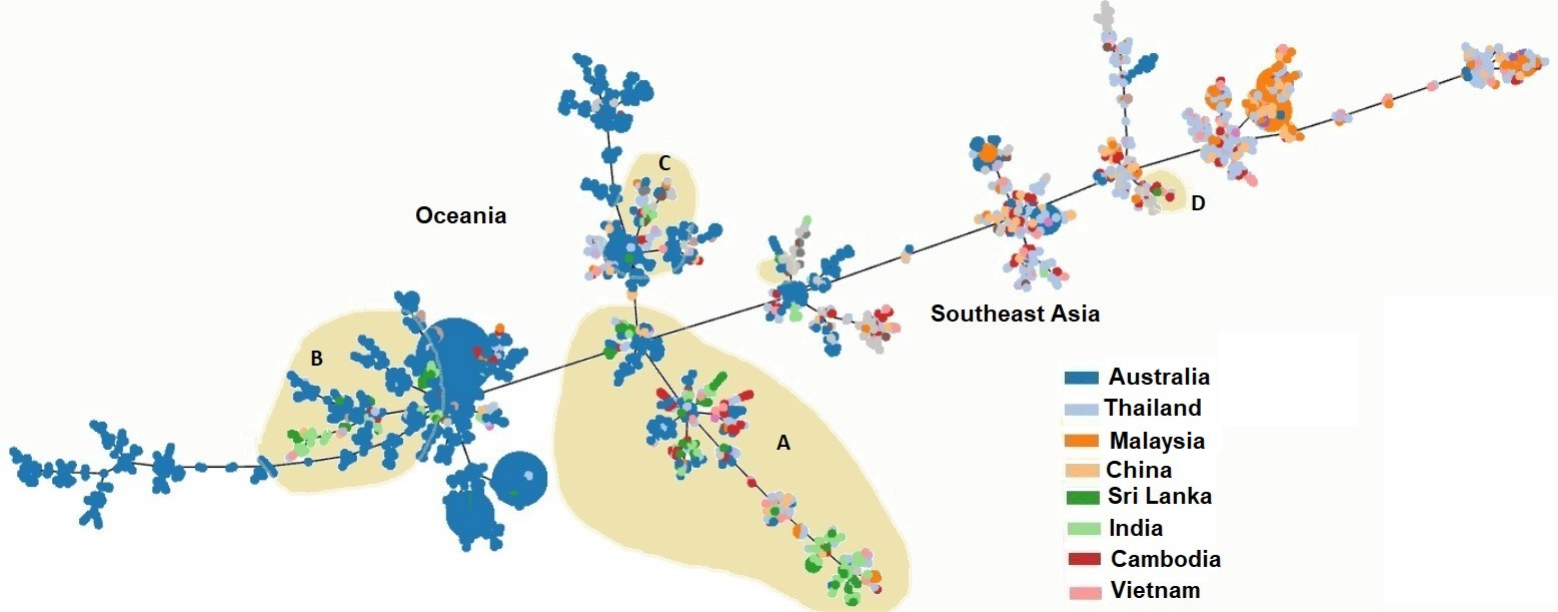
PHYLOVIZ analysis showing the genetic relationship among global collection of sequence types (STs) of *Burkholderia pseudomallei* reported from Thailand (ash), Malaysia (orange), China (light orange), Sri Lanka (dark green), India (light green), Cambodia (red) and Vietnam (pink). Sri Lankan STs (shaded in beige) clustered in four groups (A, B, C and D). Group A and D clustered with STs from Southeast Asia, group B and C clustered with STs from Oceania.

PHLOVIZ analysis showed that two distinct clades are formed by *B*. *pseudomallei* from Oceania and Southeast Asia. Sri Lankan STs (shaded in beige color) fall into four groups; A, B, C and D (Fig 5). Majority of Sri Lankan STs were clustered in Group A, B and C. Group A and D clustered with STs from Southeast Asia while group B and C clustered with STs from Oceania. Therefore, the phylogenetic relationship of Sri Lankan STs shows that they are intermediate to Australian and Southeast Asian *B*. *pseudomallei* populations.

**Fig 6:**
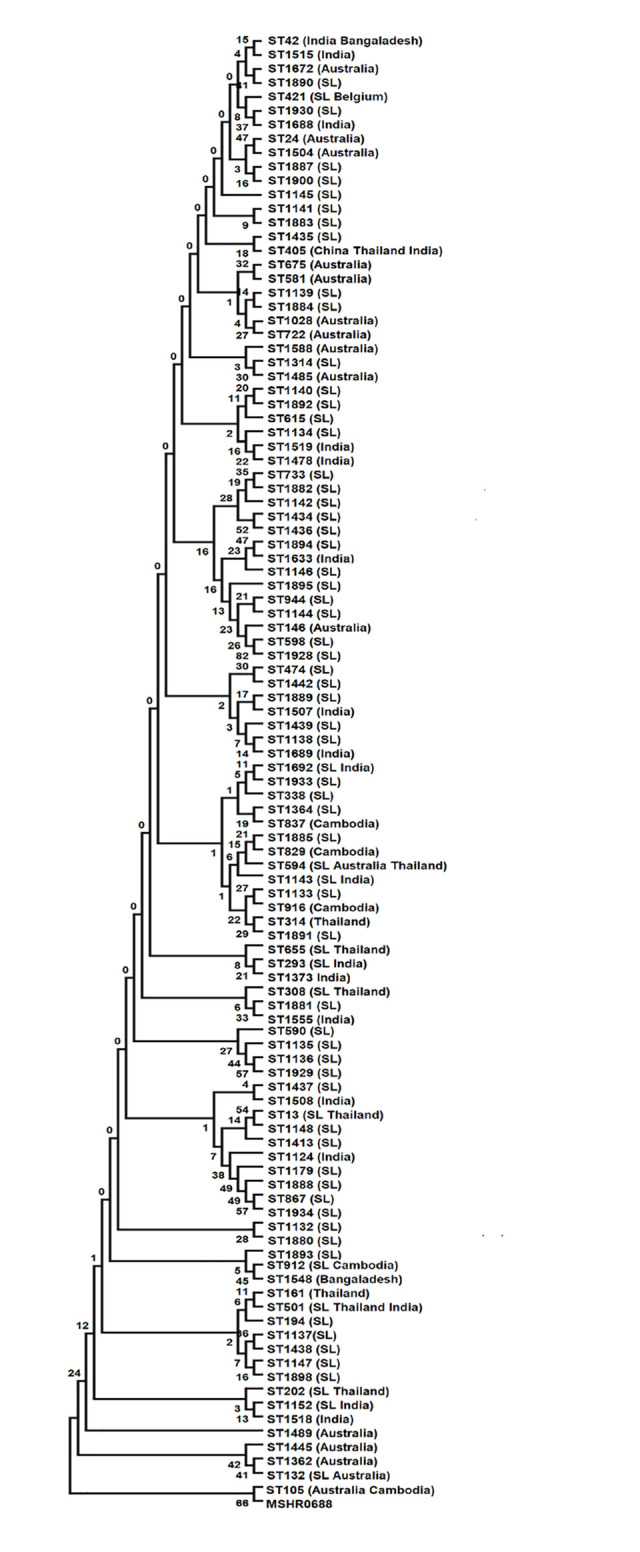
Minimum spanning tree showing the evolutionary relationship of 107 known STs including all 71 STs reported from Sri Lanka inferred using the Maximum likelihood method. Tree constructed from the concatenated sequences of the seven MLST loci. The optimal tree with the sum of branch length = 0.0378947.

To ascertain the evolutionary relationship of Sri Lankan STs, Maximum Likelihood evolutionary tree (ME) was constructed using concatenated sequences of all seven housekeeping loci (Fig 6). *B*. *pseudomallei* MSR0688 was used as an outgroup. In ME analysis, the phylogenetic relatedness of all 17 singletons to the other Sri Lankan STs, which was not resolved by the e-BURST algorithm, was observed. For example, singletons ST1883 and ST1141 clustered together and singleton ST1887 clustered with Sri Lankan Group 4 ST1900. However, some singletons clustered with STs from other endemic regions such as singleton ST293 which clustered with the Indian ST1373. The singleton ST1133 was found to be phylogenetically close to Cambodian ST916. Further, singletons ST 308, ST1141, ST1881, ST1883 and Indian ST1555 formed a separate cluster in evolutionary analysis suggesting a common evolutionary linkage among those isolates. Furthermore, the shared ST202 (Sri Lanka and Thailand) was phylogenetically close to the shared ST1152 (Sri Lanka and India) and Indian ST1518 forming a single cluster. Several novel STs (ST1890, ST1881, ST1930) were found to have phylogenetic relatedness with STs from Australia and India (marked with parenthesis) and may represent recent importation. The measure of genetic differentiation (F_ST_) for the Sri Lankan population was found to be 0.00139.

## Discussion

*B*. *pseudomallei* is an important cause of community acquired pneumonia and septicemia in endemic countries. The risk of infection is increased after flooding and a case cluster was reported in eastern Sri Lanka in 2015 following heavy rainfall [24]. Risk groups in Sri Lanka include rice farmers and rural populations engaged in subsistence cultivation in home gardens. Nationwide surveillance has been carried out since 2006 and the state public health system offers free diagnostics and free antibiotic therapy. The incidence of melioidosis in Sri Lanka has increased in tandem with increased awareness among clinicians [5].

MLST is a widely used epidemiological tool to characterize the genetic diversity of B. *pseudomallei* within and across endemic regions [2,13,32]. The data generated using MLST can be further analyzed using phylogenetic tools such as e-BURST and PHYLOVIZ to elucidate the genetic relatedness of *B*. *pseudomallei* from distinct locations [26]. ME analysis based on concatenated nucleotide sequences of housekeeping loci may be used to further resolve the genetic relationship of *B*. *pseudomallei* strains. In addition, RT-PCR to detect *v*ariably present genetic markers such as YLF/BTFC gene clusters, bimA_BM_/bimA_BP_, fhaB3 and LPSA in *B*. *pseudomallei* have been used to elucidate strain origin, genetic relatedness and virulence [33,7]. Therefore, genotyping of these four selected genetic markers, along with MLST, may provide improved resolution of genetic diversity among *B*. *pseudomallei* across the country.

*The* YLF/BTFC gene clusters are mutually exclusive in the *B*. *pseudomallei* genome and is highly correlated with specific geographic regions with BTFC strains rarely found in Southeast Asian countries [21]. YLF gene cluster is known to be prevalent in Southeast Asia including Thailand (98%) and is uncommon in Australia (12%). Whereas BTFC is the most frequent genotype reported in Australia (79–88%) and is rarely found in Southeast Asia including Thailand (2%) [33]. Results of RT-PCR based genotyping showed that approximately 15% of Sri Lankan *B*. *pseudomallei* strains were BTFC. Therefore, it seems that the population in Sri Lanka comprises a mixed population of strains, intermediate between Laurasia and Oceania. BTFC clade is predominantly present in the Eastern Province (36.17% of local isolates). The reason for this regional distribution is poorly understood but it is interesting to note that the geological formation of this area (Eastern Province, 7.7853° N, 81.4279° E), known as the Vijayan Complex, is different to the rest of the country [34]. The Vijayan Complex is a distinct geological formation comprising Precambrian high-grade metamorphic rock (a composite unit of metamorphic rocks, migmatites and granites) that has undergone changes due to the climatic conditions of the dry zone which is reflected in its soil pattern and groundwater conditions.

Around 12% of the *B*. *pseudomallei* population in Australia contains *B*. *mallei*–like sequence variation of *bimA*, *bimA*_BM_, but it is rarely found in other endemic regions in Southeast Asia. For example, prevalence of *bimA*_BM_ for isolates in Thailand is 0%. Interestingly, we found that 18.5% of the Sri Lankan *B*. *pseudomallei* population contains the *bimA*_BM_ allele variant similar to that observed in the Australia. This is further evidence of a population intermediate between Laurasia and Oceania. A strong association between isolates that carry *bimA*_BM_ variant and neurological melioidosis has been reported in northern Australia and India [7,8,35]. We also found a significant (*P* = 0.019) association between *bimA*_BM_ genotype and neurological melioidosis, although this genotype comprised only 57 isolates. In contrast to the findings of Webb et al. [33], in this collection of Sri Lankan isolates the proportion of the *bimA*_BM_ genotype among the LPSA positive strains was not different from that among LPSA negative strains (49/251, 20% and 4/42, 17% respectively. The majority of Sri Lankan clinical *B*. *pseudomallei* isolates possessed the *fhaB3* variant (80.79%) and the LPSA gene cluster (86.57%,). The *fhaB3* variant is found in 100% of Thai strains and 83% of Australian strains and LPSA is found in 97% of Thailand strains and 87% of Australian strains again suggesting a population intermediate between Laurasia and Oceania [22,33].

Considerable genetic diversity was observed among the 193 Sri Lankan clinical isolates of *B*. *pseudomallei* with multiple groups and sub-group founders within the clonal complex and singletons. Isolates with common STs were genetically diverse in terms of YLF/BTFC, *bimA*, *fhaB3 and LPSA*. For example, clinical isolate BPs 206 and BPs14 which carry the *bimA*_BM_ allele share ST1136 with BPs 211, 179, 173, 164, 158, 148 which carry *bimA*_BP_. The majority of ST1136 isolates carried *fhaB3*, however BPs 14 and BPs179 did not possess this allele type. In contrast, genotyping of ST1132 (n = 16) ST1136 (n = 16) and ST1137 (n = 35) showed that all these isolates belonged to the YLF-group and contained *bimA*_BP_ (except two isolates of 1136), *fhaB3* (except 9 isolates) and LPSA (except 6 isolates).

Several shared STs were observed with Australia, Thailand, Cambodia, India and Belgium. As observed previously, this could be due to homoplasy [34]. This demonstrates the limitation of the use of MLST for highly recombinogenic bacteria like *Burkholderia* species. Hence, analysis of whole genome data is important [36]. Migration of humans and animals might also have resulted in the dissemination of shared STs among these geographical locations [2].

PHYLOVIZ analysis has identified two distinct clades of *B*. *pseudomallei* in Oceania and Southeast Asia which could reflect geographical isolation over a long period of time [2,15,17]. However, some mixing of strains through exchange of flora and fauna between Australia and Southeast Asia may have occurred via transient land links [2]. ME analysis also supported the assumption that Sri Lankan *B*. *pseudomallei* genotypes are in between Oceania and Southeast Asia.

Phylogenetic analysis in this study revealed that Sri Lankan strains are intermediate to Australian and Southeast Asian *B*. *pseudomallei* populations. The relationship between Sri Lankan and Australian strains could date back to the period when they were linked in Gondwanaland [37]. However, the presence of several STs with phylogenetic relatedness with STs from India, Bangladesh and Cambodia may be partly attributable to multiple introductions of *B*. *pseudomallei* through anthropogenic sources, especially travel and trade routes [2,16].

## Conclusion

This is the first study that describes the genetic diversity of the Sri Lankan *B*. *pseudomallei* population using MLST along with genotyping of 4 selected genetic markers and provides a better insight into the genetic diversity and biogeography of *B*. *pseudomallei* clinical strains. Considerable genetic diversity is observed among the *B*. *pseudomallei* population in Sri Lanka in terms of both STs and the other biomarkers. The *B*. *pseudomallei* population in Sri Lanka appears to be intermediate between Southeast Asia and Oceania in terms of the prevalence of YLF/BTFC, *bimA*_BP_/*bimA*_BM_ and *fhaB3*. This may relate to its ancient position in Gondwanaland adjacent to Oceania. In contrast, several STs showed phylogenetic relatedness with STs from India, Bangladesh and Cambodia indicating the possibility of introduction of these STs on subsequent occasions.

In conclusion, this study demonstrates the usefulness of high-resolution molecular typing by including additional genetic markers along with the MLST to enhance the resolution of genetic diversity among clinical isolates to elucidate the true relationship of such isolates to each other. Further, genetic markers could help to locate the Sri Lankan isolates within the broad geographical boundaries of *B*. *pseudomallei* at a global level.

## Supporting information

S1 Table Clinical presentations of melioidosis (n = 310). (PDF)

S2 Table Geographic distribution of melioidosis cases reported during 2006 to 2018 in Sri Lanka. (PDF)

S3 Table Genetic diversity, regional distribution, clinical presentation and clinical outcome of patients with *Burkholderia pseudomallei* harboring the *bimA*_BM_ variant (n = 57). (PDF)

S4 Table Multilocus sequence typing of 84 isolates. (PDF)

S5 Table Genetic diversity, geographic distribution and clinical outcomes of melioidosis patients with *Burkholderia pseudomallei* that shared the genotype ST1132. (PDF)

S6 Table Genetic diversity, geographic distribution and clinical outcome of melioidosis patients with *Burkholderia pseudomallei* that share the commonest genotype ST1137. (PDF)

S7 Table Regional distribution of the most common STs in Sri Lanka. (PDF)
