# Supplementary material for: Biogeography and genetic diversity of clinical isolates of Burkholderia pseudomallei in Sri Lanka
Source: PLoS Negl Trop Dis. 2021 Dec 1;15(12):e0009917. doi: 10.1371/journal.pntd.0009917 (PMC8824316; doi:10.1371/journal.pntd.0009917)
Supplement: S1 Table — (PDF) [file pntd.0009917.s001.pdf]

S1 Table. Clinical presentations of melioidosis (n=310)

Clinical presentation	Number	Percentage (%)
Sepsis	198	63.8
Abscess	150	48.4
Pneumonia	89	28.7
Septic arthritis	47	15.2
Neurological melioidosis	11	3.5
Other (urinary, bone, cellulitis, endocarditis, prostatitis, sinusitis, etc.,)	64	20.6
