# Supplementary material for: Biogeography and genetic diversity of clinical isolates of Burkholderia pseudomallei in Sri Lanka
Source: PLoS Negl Trop Dis. 2021 Dec 1;15(12):e0009917. doi: 10.1371/journal.pntd.0009917 (PMC8824316; doi:10.1371/journal.pntd.0009917)
Supplement: S2 Table — (PDF) [file pntd.0009917.s002.pdf]

S2 Table. Geographic location of *Burkholderia psedomallei* clinical isolates reported from 2006 to 2018 in Sri Lanka with distribution of YLF / BTFC gene clusters

Province	*YLF or BTFC	GPS coordinates (latitude, longitude)
CP	BTFC	7.32286, 80.65913
	YLF	7.29057, 80.63372;7.33577, 80.47115;7.21531, 80.59799;7.51994, 80.9296;7.33257, 80.42945;7.2679, 80.69233;7.44933, 80.94077;7.80164, 80.6675;7.18618, 80.60632;7.37921, 80.59105;
UP	BTFC	7.46462, 81.01955
	YLF	6.88188, 81.08349;7.33161, 81.00368;6.43975, 81.13339;7.33162, 81.00371;7.1624, 81.22158;7.46336, 81.01805;7.00032, 81.04222
NWP	BTFC	7.48176, 80.36088; 7.37062, 79.83527; 7.68448, 79.926; 7.77836, 79.94879; 7.65505, 80.12638; 7.35628,79.87239; 7.4175, 79.81636; 7.68439, 79.92579; 7.51774, 80.33364; 7.4924, 79.91087; 7.98649,80.28787; 7.59822, 79.93721; 7.57059, 79.82687
	YLF	80.15886;7.67242, 80.41596; 6.63074, 80.00458; 7.46171, 80.48201; 7.80692, 80.07895;7.52771, 79.82127;7.74638, 80.13169;7.50201, 80.17376; 7.72738, 80.26442;7.56154, 80.36864;7.87808, 80.01142;7.2657, 79.85912;7.41248, 79.85908;7.32954, 80.02275; 7.58338, 79.85908;7.43747, 80.33815;7.48583, 79.8775;7.68439, 79.92579;7.4175, 79.81636;7.42167, 80.32966;7.81939, 80.27115; 7.36785, 80.06746;7.45964, 80.29484;7.77828, 79.94864; 7.74416, 80.44516;7.32748, 80.29345;7.43251, 80.21539;7.41971, 80.32673; 7.47985, 80.36224;7.43292, 80.21637;7.75186, 80.31156;7.43185, 80.21585;7.43249, 80.21663;7.29746, 79.88218;7.29543, 80.23662; 7.65513, 80.1261;7.43207, 80.2168;7.74638, 80.13169;7.5022, 80.34822;7.39633, 80.26483;7.80378, 79.82245;7.71847, 80.15487; 7.74642, 80.13178;7.58221, 80.33523;7.3879, 80.30148;7.51267, 80.2164;7.74638, 80.13169; 7.53585, 80.27862
EP	BTFC	7.78598, 81.5901; 7.52937, 81.7946; 7.58799, 81.78041;7.68555, 81.72657; 7.52993, 81.79832; 7.47776, 81.63638; 7.68521, 81.7264; 7.21958, 81.85159; 7.57963, 81.78461; 7.58131, 81.78583; 7.68516, 81.72692; 7.67127, 81.04648; 7.56666, 81.68333; 8.4579, 81.2684; 7.41438, 81.83063; 7.39113, 81.7322; 7.51916, 81.76913
	YLF	7.30175, 81.67472;7.30176, 81.67473;7.53111, 81.34544;7.37524, 81.73251;7.65695, 81.74041;7.68536, 81.72601;7.52401, 81.76601; 7.57529, 81.75356; 7.43501, 81.62912;7.53111, 81.34544;7.77689, 81.6042;7.18419, 81.67472;7.21938, 81.84976;7.21939, 81.84975; 7.21939, 81.84975;7.35748, 81.7951;7.68536, 81.72601;7.21939, 81.84975;7.62439, 81.55284;7.43488, 81.63638;7.20035, 81.83064; 7.21958, 81.85074;7.73099, 81.67472;7.21932, 81.85031;7.35315, 81.85662;7.21939, 81.84975;7.21938, 81.84976;7.21941, 81.85228; 7.70074, 81.53838;7.67127, 81.04648
NCP	BTFC	7.76984, 81.17579;8.25437, 80.63659;

	YLF	8.0421, 80.59383;7.90497, 81.13154;8.15053, 80.97898;7.93965, 81.01897;7.85934, 81.10305;7.94596, 81.24912;7.94596, 81.24912; 7.76934, 81.17474;7.73438, 80.79347;8.83409, 80.76073;7.78568, 80.53295;8.31135, 80.40365
SP	BTFC	6.31936, 81.00236;6.09216, 80.19029;6.1225, 80.73827
	YLF	7.4859, 79.80305;7.48176, 80.36088;7.32748, 80.29345;7.89498, 79.83527;7.89498, 79.83527;7.87777, 80.01111;7.58338, 79.85908; 7.59822, 79.93721;7.57242, 79.90527;7.82115, 80.40622;7.76713, 80.24988;7.29746, 79.88218;7.73722, 79.96962; 7.32049, 80.09539; 7.45964, 80.29484; 7.89498, 79.83527;7.47212, 80.04462; 7.65673, 80.36941;7.47212, 80.04462;7.82087, 80.27181;7.79606, 80.30738; 7.42506, 80.15886;7.67242, 80.41596; 6.63074, 80.00458; 7.46171, 80.48201;7.80692, 80.07895;;7.52771, 79.82127;7.74638, 80.13169; 7.50201, 80.17376; 7.72738, 80.26442;7.56154, 80.36864;7.87808, 80.01142;7.2657, 79.85912;7.41248, 79.85908;7.32954, 80.02275; 7.58338, 79.85908;7.43747, 80.33815;7.48583, 79.8775;7.68439, 79.92579;7.4175, 79.81636;7.42167, 80.32966;7.81939, 80.27115; 7.36785, 80.06746;7.45964, 80.29484;7.77828, 79.94864; 7.74416, 80.44516;7.32748, 80.29345;7.43251, 80.21539;7.41971, 80.32673; 7.47985, 80.36224;7.43292, 80.21637;7.75186, 80.31156;7.43185, 80.21585;7.43249, 80.21663;7.29746, 79.88218;7.29543, 80.23662; 7.65513, 80.1261;7.43207, 80.2168;7.74638, 80.13169;7.5022, 80.34822;7.39633, 80.26483;7.80378, 79.82245;7.71847, 80.15487; 7.74642, 80.13178;7.58221, 80.33523;7.3879, 80.30148;7.51267, 80.2164;7.74638, 80.13169; 6.33396, 80.02777; 6.04135, 80.39616
NP	BTFC	9.67835, 80.18053; 8.88589, 79.98361
	YLF	9.26709, 80.81424; 9.68449, 80.22201; 9.38243, 80.38021; 8.75434, 80.47158; 9.38028, 80.37699; 9.50338, 80.21094; 8.85636, 80.20424; 9.38028, 80.37699; 9.26709, 80.81424; 7.74638, 80.13169; 8.7542, 80.49824
WP	BTFC	7.20323, 79.86649;7.14063, 79.90136;6.94499, 80.24354
		6.8929,79.97242;7.17248, 79.88534;7.24375, 80.12864;7.13542, 79.88851;6.71474, 79.98974;7.23852, 79.88276;7.06239, 79.96683; 7.23031, 80.01647;7.14236, 80.10377;6.50957, 79.99571;6.87231, 80.00038;6.87231, 80.00038;7.02827, 79.91779;7.14397, 80.10212; 6.59165, 80.0926;6.80175, 79.92273;6.95178, 79.91329; 6.93451, 79.90597; 6.996, 79.88487; 6.80175, 79.92273;6.95106,80.0161;6.95178, 79.91329;7.06679, 80.01199;6.92707, 79.86124; 6.99066, 79.89317; 6.99066, 79.89317;6.97167, 79.87868;6.95178, 79.91329;6.74763, 80.10262;6.89058, 79.92491;6.71063, 79.90742;6.98636, 79.90744; 6.7144, 79.98906;6.97239, 79.94754;6.99151, 79.89459; 6.52194, 80.11368;6.88676, 79.91871;6.99054, 79.89295;6.95178, 79.91329; 6.85109, 79.9212;7.30524, 80.1289;7.20079, 79.87367;7.14236, 80.10377;7.11005, 80.11415;6.90607, 79.96962;7.04837, 79.89766; 7.1842, 79.95004;7.1842, 79.95004;7.21049, 79.8809;6.95907, 79.96554;6.99193, 79.89545;6.93529, 79.88078;7.02566, 79.89338;

	YLF	7.21049, 79.8809;6.99117, 79.89193;6.98636, 79.90744;7.14236, 80.10377;6.87586, 79.93919;6.93727, 79.87175;6.89799, 79.92228; 7.2435, 80.12855;6.84327, 80.00318;6.54227, 80.1575;6.99057, 79.89433;7.24802, 79.89936;6.62914, 80.02345;6.59198, 80.09187; 7.1842, 79.95004;6.95111, 79.87407;6.99159, 79.89356;6.99534, 79.88461;7.2435, 80.12855;6.85103, 79.92046;7.15405, 80.05937; 6.91537, 80.03393;5.97397, 80.36215;7.028, 79.923;6.63628, 79.95284; 6.89783, 80.08142
SGP	BTFC	6.31623, 80.84331
	YLF	7.24239, 80.36447; 7.06751, 80.20703;7.23084, 80.26;7.18509, 80.21623; 7.31352, 80.38813;6.66686, 80.7048;

NP-Northern Province, NCP-North Central Province, UP-Uva Province, SGP-Sabaragamuwa Province, EP-Eastern Province, NWP-North Western Province, WP-Western Province, SP-Southern Province, CP-Central Province; *mutually exclusive gene clusters (YLF/BTFC), YLF – Yersinia-like fimbrial gene cluster (YLF-clade), BTFC - *Burkholderia thailandensis* flagellum and chemotaxis gene cluster (BTFC-clade),
