# Supplementary material for: Biogeography and genetic diversity of clinical isolates of Burkholderia pseudomallei in Sri Lanka
Source: PLoS Negl Trop Dis. 2021 Dec 1;15(12):e0009917. doi: 10.1371/journal.pntd.0009917 (PMC8824316; doi:10.1371/journal.pntd.0009917)
Supplement: S3 Table — (PDF) [file pntd.0009917.s003.pdf]

S3 Table. Genetic diversity, regional distribution, clinical presentation and clinical outcome of patients with *Burkholderia pseudomallei* harboring the *bimA*$_{BM}$ variant (n = 57)

Isolate	ST	YLF/BTFC	*fhab3*	LPSA	Province	Outcome	Clinical presentation
BPs3	1134	YLF	+	+	NWP	death	septic arthritis/ encephalitis/ sepsis
BPs12	1138	BTFC	+	+	Uva	death	liver abscess/ splenic abscess
BPs14	1136	YLF	-	+	NCP	paraplegia	psoas abscess / transverse myelitis
BPs 27	1148	YLF	+	+	Uva	recovered	pneumonia/ liver abscess / pleural effusion
BPs39	1145	YLF	+	+	NWP	recovered / relapsed	intramuscular abscess with osteomyelitis of L
BPs52	194	YLF	+	+	WP	recovered	sepsis
BPs71	1434	YLF	+	+	SP	recovered	sepsis, abscess in R/thigh and forearm, septic arthritis L knee,
BPs79	598	YLF	+	+	EP	recovered /relapsed	sepsis, back abscess/ arthritis of right hip
BPs80	1143	BTFC	+	+	EP	recovered	sepsis, leg abscess, necrotising fasciitis, lung abscess
BPs95	1434	YLF	-	nd	NCP	death	sepsis, brain stem encephalitis
BPs111	1364	YLF	+	-	EP	recovered /relapsed in Sep16	sepsis, septic arthritis of left elbow
BPs112	1442	YLF	+	+	EP	recovered	sepsis
BPs114	594	BTFC	+	+	EP	recovered	sepsis, fever, L/maxillary sinusitis with middle turbinate necrosis
BPS116	1179	BTFC	+	+	EP	death	sepsis, broncho pneumonia multiple pustules in lung, liver and splenic abscess
BPs119	1152	BTFC	+	+	SP	recovered	fever, liver abscess
BPs121	13	YLF	+	+	EP	recovered	sepsis 52
BPs122	594	BTFC	+	-	EP	death	sepsis, bronchopneumonia, septic arthritis of knee joint, pustules on lung, liver and spleen,
BPs128	1928	YLF	+	+	EP	recovered	fever, submandibular abscess
BPs139	1887	BTFC	+	+	EP	recovered	splenic abscess
BPs151	1890	BTFC	+	+	EP	recovered	sepsis
BPs154	501	YLF	+	+	NCP	recovered	sepsis

BPs161	1893	BTFC	+	+	NP	death	septic shock, cough, dysuria, diarrhoea, splenic abscess
BPs162	1889	BTFC	+	+	NP	recovered	sepsis, pneumonia, skin and soft itssue abscess, septic arthritis
BPs175	202	YLF	+	+	NP	death	liver, spleen, lung abscess
BPs184		YLF	+	+	SGP	recovered	sepsis, pneumonia, multiple skin abscess
BPs189	1895	YLF	-	+	NP	recovered	neutropenic sepsis
BPs190	1900	YLF	+	+	NP	death	sepsis, pneumonia, chest wall abscess, septic arthritis
BPs205	1933	YLF	+	+	NP	recovered	parotid abscess
BPs206	1136	YLF	+	+	NCP	recovered	splenic abscess
BPs220	nd	BTFC	+	+	NWP	recovered	cellulitis of the foot
BPs222	nd	BTFC	+	+	WP	recovered	sepsis
BPs223	nd	BTFC	+	+	EP	recovered	sepsis
BPs224	nd	YLF	+	+	EP	recovered / relapse	suprapatellar bursitis, septic arthritis
BPs237	nd	BTFC	+	+	EP	recovered	splenic abscess, abscess hand and leg
BPs244	nd	YLF	+	+	EP	LAMA	sepsis
BPs245	nd	YLF	+	+	EP	recovered	sepsis, septic arthritis of R/KJ, acute kidney injury
BPs246	nd	BTFC	+	+	NCP	recovered	bilateral inguinal abscess
BPs247	nd	BTFC	+	+	EP	death	sepsis, bilateral pleural effusion, myocarditis, joint swelling
BPs256	nd	YLF	+	-	WP	death	sepsis, severe cavitating pneumonia, pyelonephritis
BPs257	nd	YLF	+	+	EP	recovered	
BPs258	nd	YLF	+	+	EP	recovered	pneumonia
BPs259	nd	YLF	+	+	EP	recovered	sepsis, septic arthritis
BPs262	nd	YLF	+	-	EP	paraplegia	intramedullary spinal SOL
BPs263	nd	YLF	+	+	SP	recovery	pneumonia
BPs269	nd	YLF	+	+	NCP	death	cellulitis
BPs271	nd	YLF	+	+	SP	death	pneumonia, cellulitis
BPs281	nd	YLF	-	+	SP	death	pneumonia, pleural effusion

BPs289	nd	YLF	+	+	WP	death	Sepsis, pyelonephritis
BPs296	nd	YLF	-	+	SP	death	cellulitis of left leg, pneumonia
BPs309	nd	BTFC	+	+	EP	recovered	meningoencephalitis, jaw abscess
BPs311	nd	YLF	-	+	EP	death	pneumonia, myocarditis
BPs312	nd	YLF	+	+	EP	death	pneumonia, septic shock
BPs317	nd	YLF	+	-	NP	death	septic shock, splenic abscess, liver abscess, suprarenal abscess, chest abscess
BPs318	nd	YLF	-	-	EP	death	septicaemia, liver abscess, splenic abscess, septic arthritis
BPs320	nd	YLF	+	+	NP	recovered	Lung abscess
BPs327	nd	BTFC	-	-	EP	death	B/L Calf carbuncles, B/L septic arthritis of knee, abscess
BPs328	nd	BTFC	-	+	EP	recovered / relapse	Multiple subcutaneous abscesses /cellulitis

UVA - Uva Province, NWP - North Western Province, CP - Central Province, WP - Western Province, SP - Southern Province, NCP - North Central Province, SGP - Sabaragamuwa Province, $bimA_{BP}$/$bimA_{BM}$ – *Burkholderia* intracellular motility factor (*BimA*) gene variants, *fhaB3* - filamentous hemagglutinin 3, YLF – *Yersinia*-like fimbrial gene cluster (YLF-clade), BTFC - *Burkholderia thailandensis* flagellum and chemotaxis gene cluster (BTFC-clade), LPSA - lipopolysaccharide (LPS) O-antigen type A, + - positive, – - negative, nd - not determined
