# Supplementary material for: Biogeography and genetic diversity of clinical isolates of Burkholderia pseudomallei in Sri Lanka
Source: PLoS Negl Trop Dis. 2021 Dec 1;15(12):e0009917. doi: 10.1371/journal.pntd.0009917 (PMC8824316; doi:10.1371/journal.pntd.0009917)
Supplement: S4 Table — (PDF) [file pntd.0009917.s004.pdf]

S4 Table. Multilocus sequence typing of 84 Sri Lankan clinical isolates

PubMLST strain id (http://pubmlst.org/bpseudomallei.mlst.net/)	Allelic profiles							Sequence type
	ace	*gltB*	*gmhD*	*lepA*	*lipA*	*narK*	*ndh*	
6414	1	2	6	4	1	2	3	1884
6415	1	4	3	2	1	61	1	1885
6417	4	4	13	4	1	2	1	1887
6418	4	4	10	2	1	42	1	1888
6419	1	4	3	4	1	1	1	1889
6420	4	12	6	19	1	2	1	1890
6421	1	4	3	1	1	2	3	1891
6422	4	6	6	2	1	1	3	1892
6423	4	2	6	2	1	2	1	1893
6424	1	4	3	4	1	1	1	1889
6425	1	2	3	2	1	60	1	1883
6426	1	12	6	2	5	8	3	1882
6427	4	2	3	4	20	2	3	1895
6428	1	1	10	3	1	1	3	1881
6429	1	12	6	2	1	60	3	1880
6430	1	2	6	2	20	8	3	1894
6431	1	12	6	4	1	8	3	1898
6432	4	2	13	2	1	2	1	1900
6433	1	2	3	4	1	2	57	590
6434	1	4	3	2	1	2	1	594
6435	1	4	3	2	1	2	1	594
6436	1	4	3	2	1	2	1	594
6437	4	4	3	4	3	2	20	1928
6438	1	2	6	46	1	2	57	1929
6439	1	12	6	2	20	1	3	1146
6440	1	12	14	4	1	1	3	1930
6441	1	2	6	2	1	8	3	1137
6442	4	2	6	2	1	1	3	1140
6443	4	2	6	2	1	1	3	1140
6444	4	12	3	4	25	2	3	421
6445	1	2	6	4	1	2	57	1135
6446	18	4	3	4	1	1	1	1439
6447	1	2	6	2	1	8	3	1137
6448	1	12	6	2	1	1	3	1132
6449	1	2	6	2	1	2	57	1136
6450	1	2	6	2	1	8	3	1137
6451	4	2	6	2	1	1	3	1140
6452	1	2	6	2	1	2	57	1136
6453	1	12	6	2	20	1	3	1146
6454	4	2	6	2	1	1	3	1140

6455	1	12	6	2	1	2	3	501
6456	1	12	6	2	1	1	3	1132
6457	8	2	6	4	1	2	3	1139
6458	1	2	6	2	1	2	57	1136
6459	4	2	6	2	1	1	3	1140
6460	1	2	6	2	1	8	3	1137
6461	1	2	6	2	1	2	57	1136
6462	1	2	6	2	1	8	3	1137
6463	1	4	3	2	1	2	3	1143
6464	8	2	6	4	1	2	3	1139
6465	1	4	3	2	1	1	3	1692
6466	1	12	6	2	1	1	3	1132
6467	8	2	6	4	1	2	3	1139
6468	1	2	6	2	1	2	57	1136
6469	1	2	14	2	5	2	1	1434
6470	4	2	3	4	1	2	1	202
6471	1	2	6	2	1	8	3	1137
6472	1	4	3	2	1	1	1	1364
6473	1	2	6	2	1	2	57	1136
6474	1	12	6	2	1	1	3	1132
6475	1	2	6	2	1	8	3	1137
6476	8	2	6	4	1	2	3	1139
6477	1	2	14	2	5	2	1	1434
6479	1	2	6	2	1	8	3	1137
6480	1	12	6	2	1	1	3	1132
6481	1	12	6	2	1	1	3	1132
6482	1	2	6	2	1	8	3	1137
6483	1	2	6	2	1	8	3	1137
6485	4	2	6	2	1	1	3	1140
6486	1	12	6	2	1	1	3	1132
6487	1	2	6	2	1	8	3	1137
6488	1	12	6	2	20	1	3	1146
6489	1	2	6	2	1	8	3	1137
6490	1	2	6	2	1	8	3	1137
6491	1	12	6	2	1	8	3	1147
6492	1	2	6	2	1	8	3	1137
6493	1	12	6	2	1	1	3	1132
6494	1	4	3	2	1	1	87	1933
6495	1	2	6	2	1	2	57	1136
6496	4	4	10	2	1	42	11	1934
6497	1	2	6	2	1	8	3	1137
6498	1	2	6	2	1	8	3	1137
6499	1	2	6	2	1	8	3	1137
6500	1	2	6	2	1	2	57	1136
