# Supplementary material for: Biogeography and genetic diversity of clinical isolates of Burkholderia pseudomallei in Sri Lanka
Source: PLoS Negl Trop Dis. 2021 Dec 1;15(12):e0009917. doi: 10.1371/journal.pntd.0009917 (PMC8824316; doi:10.1371/journal.pntd.0009917)
Supplement: S5 Table — (PDF) [file pntd.0009917.s005.pdf]

S5 Table. Genetic diversity, geographic distribution and clinical outcomes of melioidosis patients with *Burkholderia pseudomallei* that shared the founder genotype ST1132.

	Isolate		**YLF/ BTFC clade**	***fhab3***	**$bimA_{BM}$/ $bimA_{BP}$**	**LPSA**	**Geographical region**	**Clinical presentations**
1	BPs2a/b		YLF	+	$bimA_{BP}$	+	CP	multiple skin and soft tissue abscesses, septic arthritis, sepsis
2	BPs11		YLF	+	$bimA_{BP}$	+	WP	psoas abscess
3	BPs43		YLF	+	$bimA_{BP}$	+	UVA	multiple skin and soft tissue abscesses, septic arthritis, sepsis
4	BPs45		YLF	+	$bimA_{BP}$	+	NWP	abscess over the chest, P/H of abscess over right thigh
5	BPs60		YLF	+	$bimA_{BP}$	+	NWP	sepsis/ headache / abdominal pain
6	BPs61		YLF	+	$bimA_{BP}$	+	NWP	splenic abscess, sepsis
7	BPs64		YLF	+	$bimA_{BP}$	+	NWP	R/S knee joint septic arthritis
8	BPs91		YLF	+	$bimA_{BP}$	+	NWP	endocarditis of aortic valve
9	BPs 144		YLF	+	$bimA_{BP}$	+	NWP	sepsis
10	BPs 155		YLF	+	$bimA_{BP}$	+	WP	sepsis
11	BPs 171		YLF	+	$bimA_{BP}$	+	WP	NA
12	BPs 180		YLF	-	$bimA_{BP}$	+	NWP	cervical lymph node abscess
13	BPs 186		YLF	+	$bimA_{BP}$	+	WP	sepsis, prostatitis,
14	BPs 191		YLF	-	$bimA_{BP}$	+	NWP	parotid abscess
15	BPs 196		YLF	-	$bimA_{BP}$	+	NWP	splenic abscess
16	BPs 204		YLF	+	$bimA_{BP}$	+	NWP	parotid abscess

UVA - Uva Province, NWP - North Western Province, CP - Central Province, WP - Western Province, SP - Southern Province, NCP - North Central Province, SGP - Sabaragamuwa Province, $bimA_{BP}$/$bimA_{BM}$ – *Burkholderia* intracellular motility factor (*BimA*) gene variants, *fhaB3* - filamentous hemagglutinin 3, YLF – *Yersinia*-like fimbrial gene cluster (YLF-clade), BTFC - *Burkholderia thailandensis* flagellum and chemotaxis gene cluster (BTFC-clade), LPSA - lipopolysaccharide (LPS) O-antigen type A, + - positive, – - negative, nd - not determined.
