# Supplementary material for: Biogeography and genetic diversity of clinical isolates of Burkholderia pseudomallei in Sri Lanka
Source: PLoS Negl Trop Dis. 2021 Dec 1;15(12):e0009917. doi: 10.1371/journal.pntd.0009917 (PMC8824316; doi:10.1371/journal.pntd.0009917)
Supplement: S6 Table — (PDF) [file pntd.0009917.s006.pdf]

S6 Table. Genetic diversity, geographic distribution and clinical outcome of melioidosis patients with *Burkholderia pseudomallei* that share the commonest genotype ST1137

	**Isolate**	**Region**	**Disease outcome**	**YLF/ BTFC clade**	***fhab3* variant**	***bimA*$_{BM}$/ *bimA*$_{BP}$**	***LPSA***
1	BPs8	Unknown	Death	YLF	+	*bimA*$_{BP}$	+
2	BPs9	Unknown	Survived	YLF	+	*bimA*$_{BP}$	+
3	BPs17	Unknown	Survived	YLF	+	*bimA*$_{BP}$	-
4	BPs24	Unknown	Death	YLF	+	*bimA*$_{BP}$	+
5	BPs26	Unknown	Death	YLF	+	*bimA*$_{BP}$	+
6	BPs65	NWP	Survived	YLF	+	*bimA*$_{BP}$	+
7	BPs70	WP	Survived	YLF	+	*bimA*$_{BP}$	+
8	BPs82	WP	Death	YLF	+	*bimA*$_{BP}$	+
9	BPs83	WP	Survived	YLF	+	*bimA*$_{BP}$	+
10	BPs85	WP	Survived	YLF	+	*bimA*$_{BP}$	+
11	BPs86	NCP	Survived	YLF	+	*bimA*$_{BP}$	+
12	BPs90	SGP	Death	YLF	+	*bimA*$_{BP}$	+
13	BPs92	WP	Survived	YLF	nd	nd	nd
14	BPs96	WP	Survived	YLF	+	*bimA*$_{BP}$	+
15	BPs97	SGP	Survived	YLF	+	*bimA*$_{BP}$	+
16	BPs99	WP	Survived	YLF	+	*bimA*$_{BP}$	+
17	BPs100	WP	Survived	YLF	+	*bimA*$_{BP}$	+
18	BPs120	NWP	Survived	YLF	+	*bimA*$_{BP}$	+
19	BPs 134	WP	Death	YLF	+	*bimA*$_{BP}$	-
20	BPs 142	WP	Survived	YLF	-	*bimA*$_{BP}$	+
21	BPs 146	WP	Survived	YLF	+	*bimA*$_{BP}$	+
22	BPs 160	WP	Survived	YLF	+	*bimA*$_{BP}$	+
23	BPs 165	NWP	Death	YLF	+	*bimA*$_{BP}$	+
24	BPs 176	WP	Survived	YLF	+	*bimA*$_{BP}$	+
25	BPs 181	WP	Survived	YLF	+	*bimA*$_{BP}$	+
26	BPs 185	Unknown	Death	YLF	+	*bimA*$_{BP}$	+
27	BPs 192	WP	Survived	YLF	+	*bimA*$_{BP}$	+
28	BPs 193	SP	Survived	YLF	-	*bimA*$_{BP}$	+
29	BPs 197	WP	Survived	YLF	+	*bimA*$_{BP}$	+
30	BPs 199	WP	Survived	YLF	+	*bimA*$_{BP}$	+
31	BPs 201	UVA	Survived	YLF	+	*bimA*$_{BP}$	+
32	BPs 203	SP	Survived	YLF	+	*bimA*$_{BP}$	+
33	BPs 208	WP	Survived	YLF	-	*bimA*$_{BP}$	+
34	BPs 209	WP	Death	YLF	-	*bimA*$_{BP}$	+

35	BPs 210	NWP	Survived	YLF	+	*bimA*$_{BP}$	+

UVA - Uva Province, NWP - North Western Province, CP - Central Province, WP - Western Province, SP - Southern Province, NCP - North Central Province, SGP - Sabaragamuwa Province, *bimA*$_{BP}$/*bimA*$_{BM}$ – *Burkholderia* intracellular motility factor (*BimA*) gene variants, *fhaB3* - filamentous hemagglutinin 3, YLF – *Yersinia*-like fimbrial gene cluster (YLF-clade), BTFC - *Burkholderia thailandensis* flagellum and chemotaxis gene cluster (BTFC-clade), LPSA - lipopolysaccharide (LPS) O-antigen type A, + - positive, – - negative, nd - not determined.
