# Supplementary material for: Biogeography and genetic diversity of clinical isolates of Burkholderia pseudomallei in Sri Lanka
Source: PLoS Negl Trop Dis. 2021 Dec 1;15(12):e0009917. doi: 10.1371/journal.pntd.0009917 (PMC8824316; doi:10.1371/journal.pntd.0009917)
Supplement: S7 Table — (PDF) [file pntd.0009917.s007.pdf]

S7 Table. Regional distribution of the most common STs in Sri Lanka

**Sequence type (ST)**	**No. of isolates**	**Regional distribution (Frequency)**	**Clade (YLF/BTFC)**
1137	35	CP (1), SGP (2), WP (21), NCP (2), SP (3), NWP (4), UVA (1) Unknown (1)	YLF
1136	16	NWP (6), WP (1), UVA (2), SP (1), NCP (2), CP (3), SGP (1),	YLF
1132	16	UVA (1), NWP (10), WP (4), CP (1)	YLF
1140	11	WP (8), SP (1), EP (1), NWP (1)	YLF
1135	10	NWP (6), WP (2), CP (2)	YLF
1434	8	NCP (1), SP (2), WP (4), NWP (1)	YLF
594	7	EP (4), SP (1), WP (2)	YLF-4 BTFC-3
1139	7	NWP (5), WP (2)	BTFC-6 YLF-1
1146	04	NWP (4)	YLF
1147	04	CP (1), WP (2), NWP (1)	YLF
UVA- Uva Province, NWP - North Western Province, CP - Central Province, WP - Western Province, SP - Southern Province, SGP - Sabaragamuwa Province			
